# Atypical Polyneuropathy, Organomegaly, Endocrinopathy, Monoclonal Protein, and Skin Changes (POEMS) Syndrome

**DOI:** 10.7759/cureus.103791

**Published:** 2026-02-17

**Authors:** Micheal Bishara, Anna Homeniuk, Aayush Patel, Feras Al Moussally, Venkataraman Rajagopalan

**Affiliations:** 1 Internal Medicine, University of Pittsburgh Medical Center Pinnacle, Harrisburg, USA; 2 Hematology and Medical Oncology, Drexel University College of Medicine, Harrisburg, USA; 3 Hematology and Medical Oncology, University of Pittsburgh Medical Center Pinnacle, Harrisburg, USA

**Keywords:** atypical poems, lambda-restricted plasma cell disorders, monoclonal gammopathy, monoclonal gammopathy of undetermined significance (mgus), polyneuropathy, rare case report, typical poems

## Abstract

Polyneuropathy, organomegaly, endocrinopathy, monoclonal protein, and skin changes (POEMS) syndrome is a rare paraneoplastic syndrome that occurs mostly in the context of lambda-restricted plasma cell disorders. Diagnosis is made when patients have certain disease-defining criteria. Atypical presentations of POEMS are suspected when patients do not meet those predefined diagnostic criteria.

An 82-year-old female patient was referred to the hematology clinic for persistent thrombocytosis. The patient had a history of stroke and was asymptomatic, and the clinical exam was noncontributory except for the presence of small axillary lymph nodes. Workup demonstrated a picture of an IgG kappa monoclonal gammopathy (MG), with an elevated serum free kappa light chain, a normal free lambda light chain, and an increased kappa/lambda ratio, prompting further evaluation for an underlying plasma cell disorder. Bone marrow biopsy showed plasma cell neoplasm at 20% to 30%. Both the karyotype and the plasma cell fluorescence in situ hybridization (FISH) prognosis panel were normal. Lymph node (LN) core needle biopsy showed follicular hyperplasia. Smoldering multiple myeloma (SMM) was considered.

Due to the patient’s clinical history, a diagnosis of atypical POEMS was entertained. Vascular endothelial growth factor (VEGF) was elevated. Electromyography (EMG) testing for sensory and motor conduction for lower limbs revealed abnormal findings aligning with subclinical polyneuropathy. This strongly favored the diagnosis of atypical POEMS. Initially, our patient was evaluated for an underlying plasma cell disorder, since the presence of monoclonal protein, monoclonal gammopathy of undetermined significance (MGUS), was the highest on the differential. When the bone marrow biopsy showed a 20% to 30% plasma cell infiltration, SMM was diagnosed. However, the presence of certain characteristic features for POEMS, including nodal enlargement and thrombocytosis, led to entertaining the differential diagnosis of POEMS. Markedly elevated serum VEGF levels further reinforced the diagnosis. Clinically absent neuropathy raised the concern for an atypical POEMS, but subsequently, EMG testing showed that the patient has subclinical polyneuropathy, which provided additional support for the diagnosis of atypical POEMS.

To meet the diagnosis of POEMS syndrome, challenging criteria should be fulfilled. Polyneuropathy and lambda-restricted are major criteria for the diagnosis of typical POEMS syndrome. In our case, the patient had a kappa-restricted variant and subclinical polyneuropathy, compounding this atypical POEMS presentation. This case report highlights the importance of a broad differential, including atypical presentations, while at the same time, highlighting the importance of early detection to ensure proper management for better outcomes.

## Introduction

Polyneuropathy, organomegaly, endocrinopathy, monoclonal protein, and skin changes (POEMS) syndrome is a challenging, rare, complex paraneoplastic syndrome arising from an underlying monoclonal plasma cell proliferation that mostly occurs in the context of lambda-restricted plasma cell disorders. As clinical manifestations often evolve slowly and inconsistently, early presentations may be subtle or incomplete, contributing to diagnostic delays [1].

Atypical presentation of POEMS usually refers to cases that do not fully meet all mandatory, major/minor criteria, or have subclinical or evolving features [1,2]. Atypical clinical features could include subclinical neuropathy or non‑lambda (e.g., kappa‑restricted) monoclonal proteins. Only isolated case reports have described these atypical phenotypes, and their true prevalence remains unknown. Although diagnostic uncertainty is common, outcomes are favorable when early recognition prompts appropriate therapy [1].

Given the variability in presentation and the potential for overlap with entities such as monoclonal gammopathy of undetermined significance (MGUS) and smoldering multiple myeloma (SMM), clinicians must maintain a high index of suspicion when encountering patients with unexplained monoclonal gammopathy (MG) and systemic features not fully explained by more common plasma cell disorders. This case highlights an atypical presentation of POEMS syndrome featuring kappa restriction and initially subclinical neuropathy, underscoring the importance of broad diagnostic consideration and timely evaluation.

## Case presentation

The patient was an 82-year-old female with a past medical history of obstructive sleep apnea, stroke, diabetes, hypertension, chronic kidney disease, obesity, and right bundle branch block, who was referred by her primary care provider to the hematology clinic for persistent thrombocytosis rising to above 700,000 for six consecutive months (normal range: 140-366 109/L). She was asymptomatic, with a medical history of a stroke in 2023, no family history of blood disorders, including hematological malignancies or coagulopathy, and a review of systems was unremarkable. On physical exam, a small left axillary lymph node was noted. A complete blood count (Table 1) showed normocytic anemia with a hemoglobin of 11 (11.7-15.1 gm/dl), hematocrit of 34.5% (29.4-47%), mean corpuscular volume (MCV) of 86.6 (78.9-98.6 fl), mean corpuscular hemoglobin (MCH) of 28.9 (26.8-33.8 pg), and mean corpuscular hemoglobin concentration (MCHC) of 33.4 (31.5-36.5 g/dl).

**Table 1:** Complete blood count results at presentation The complete blood count results at presentation emphasized thrombocytosis and normocytic anemia. MCV: Mean corpuscular volume, MCH: Mean corpuscular hemoglobin, MCHC: Mean corpuscular hemoglobin concentration

Parameter	Result	Units	Reference range	Interpretation
White blood cell count	6.9	×10³/µL	3.9-9.5	Within normal limits
Red blood cell count	3.98	×10⁶/µL	3.97-5.27	Within normal limits
Hemoglobin	11.5	g/dL	11.7-15.5	Mildly decreased
Hematocrit	34.5	%	34.0-47.0	Within normal limits
MCV	86.6	fL	79.0-98.0	Normocytic
MCH	28.9	pg	26.5-33.4	Within normal limits
MCHC	33.4	g/dL	31.5-36.5	Within normal limits
Red cell distribution width (coefficient of variations)	15.1	%	11.0-15.5	High-normal
Platelet count	852	×10³/µL	155-366	Markedly elevated
Mean platelet volume	6.3	fL	7.2-12.2	Decreased

Initial differential diagnoses included secondary (such as iron deficiency, inflammation, and infection) vs. primary essential thrombocytosis (ET). Workup demonstrated an IgG kappa MG, with an elevated serum free kappa light chain level of 163.5 mg/L (reference range: 3.3-19.4), a normal free lambda light chain level of 22 mg/L (reference range: 5.7-26.3), and an increased kappa/lambda ratio of 7.43 (reference range: 0.26-1.65), prompting further evaluation for an underlying plasma cell disorder.

The patient underwent a bone marrow biopsy that demonstrated plasma cell neoplasm at 20% to 30% and background trilineage hematopoiesis with increased megakaryocytes. Iron deposition was present; no ring sideroblasts were identified. The karyotype and the plasma cell fluorescence in situ hybridization (FISH) prognosis panel were normal.

Bone marrow stains that used immune stains for CD3, CD20, and CD138, and in situ hybridization for kappa and lambda light chains were performed (Table 2). The CD3 and CD20 highlighted scattered interstitial T-cells and B-cells, respectively. No lymphoid aggregates were identified. The CD138 highlighted increased plasma cells (estimated at 20% to 30% of all) that were kappa light chain restricted (kappa/lambda >10). 

**Table 2:** Flow cytometry analysis of bone marrow aspirate The table summarizes the key population analysis from the bone marrow aspiration, emphasizing the monoclonal population detected.

Cell population	Percentage of total	Phenotypic markers	Clinical interpretation
Monoclonal plasma cells	2.3%	CD19-, CD38 (bright), CD138+, CD45 (partial), CD56 (moderate)	Abnormal/clonal
Lymphocytes	25.9%	CD4:CD8 ratio of 2.3; normal phenotypic maturation	Unremarkable
Granulocytes	62.8%	Normal maturation; no dysmaturation detected	Unremarkable
Monocytes	4.0%	Co-express CD14 and CD64	Unremarkable
CD34+ Blasts	1.8%	Within normal limits	Unremarkable

**Figure 1:**
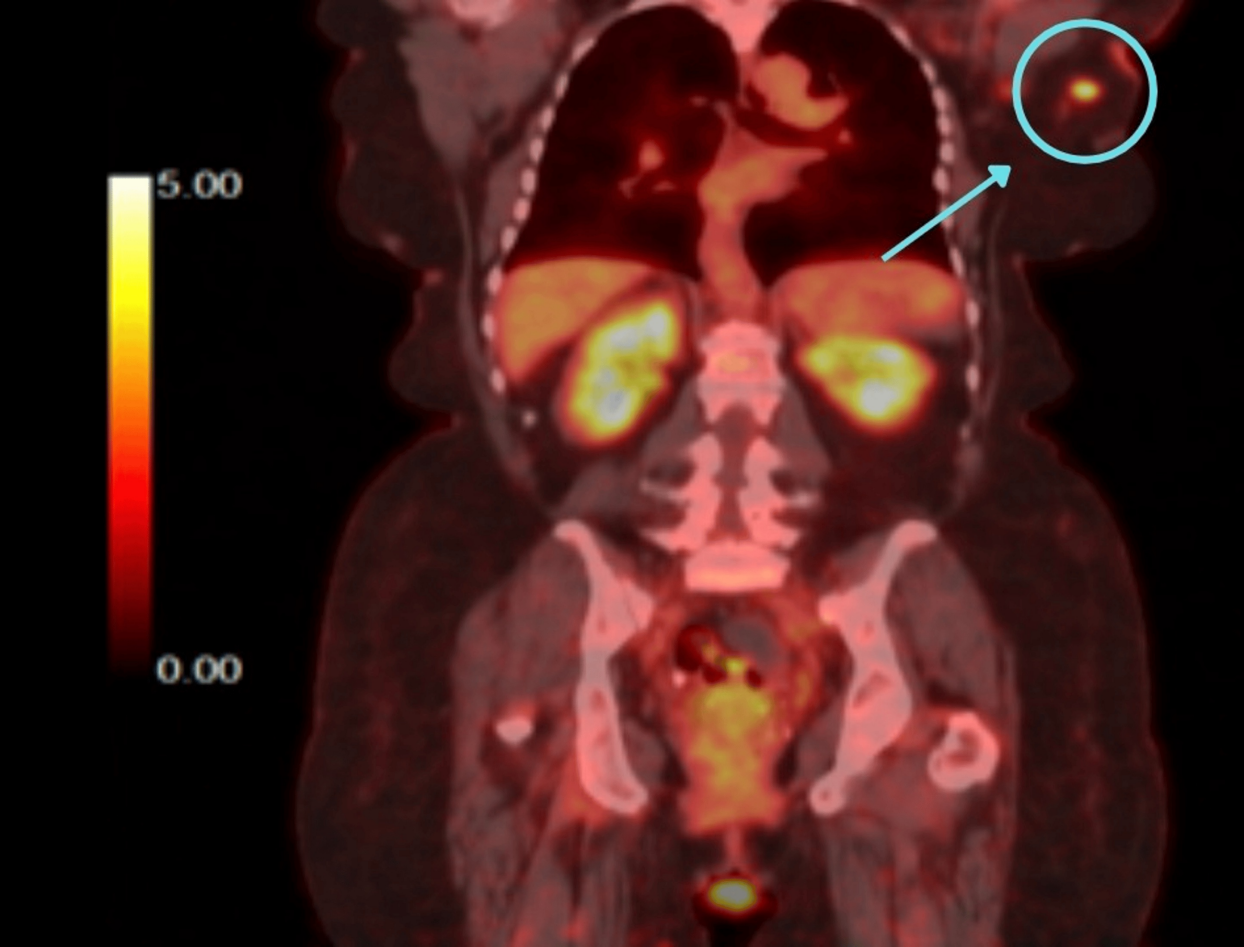
Coronal view of the PET/CT scan showing an FDG-avid left axillary node FDG: Fluorodeoxyglucose

**Figure 2:**
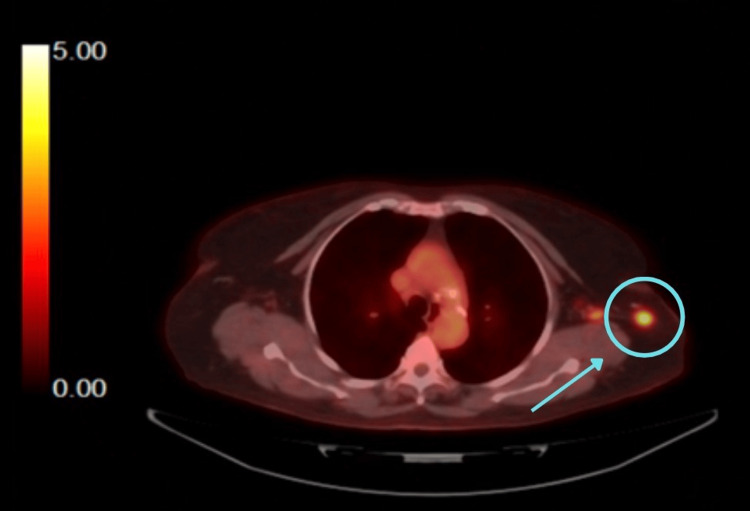
Transverse view of the PET/CT scan showing an FDG-avid left axillary node (standardised uptake value (SUV) 5.8) FDG: Fluorodeoxyglucose

The PET/CT scans (Figures 1-2) showed fluorodeoxyglucose (FDG)-avid activity in the left axillary node that was concerning for malignancy. Lymph node (LN) core needle biopsy showed reactive LN with follicular hyperplasia.

Based on the bone marrow results and clinical presentation, the patient met the criteria for MG consistent with SMM, as monoclonal protein was present in the gamma region, and the bone marrow biopsy showed more than 10% of plasma cells without symptoms or organ involvement, including bone, kidney, or hypercalcemia. Up to this point, the patient didn’t meet the full POEMS diagnostic criteria due to a lack of polyneuropathy; she only met 1/2 of the mandatory criteria (monoclonal plasma cell disorder) and 1/3 of the major criteria (elevated vascular endothelial growth factor (VEGF)), with multiple minor criteria (lymphadenopathy, thrombocytosis).

However, neuropathy can be subclinical. The patient met 3/6 of the minor criteria (edema, organomegaly, and thrombocytosis), and the diagnosis of atypical POEMS was entertained. The VEGF levels were evaluated and were markedly elevated at 832 pg/mL (normal range: 31-86 pg/mL), which is highly suggestive of POEMS. Due to the high level of suspicion, the patient was sent for electromyography (EMG) testing, which showed absent sensation in both the right sural and right superficial peroneal nerves, thus finally confirming the diagnosis of POEMS. The timeline from pathology to flow cytometry investigation of the abnormal left axillary node is summarised in Table 3.

**Table 3:** Timeline of the investigation of the abnormal left axillary node FDG: Fluorodeoxyglucose, SUV: Standardised uptake value

Test type	Date	Key finding	Clinical significance
PET/CT scan	06/27/2025	FDG-avid left axillary node (SUV 5.8)	Suspected malignancy; warrants biopsy
Pathology	08/06/2025	Benign reactive follicular hyperplasia	No evidence of lymphoma or malignancy
Flow cytometry	08/06/2025	No immunophenotypic evidence of non-Hodgkin's lymphoma	Supports the benign pathology diagnosis

## Discussion

MG and initial diagnostic considerations

Monoclonal gammopathy encompasses a spectrum of disorders characterized by excess production of abnormal monoclonal proteins (M‑antibodies) by clonal plasma cells. Clinical presentations range from completely asymptomatic MGUS to malignant entities such as multiple myeloma (MM) [1,2]. Although the precise etiology remains unclear, several risk factors have been identified, including advanced age, African American race, male sex, family history of MG, and autoimmune comorbidities. Modifiable contributors such as smoking and obesity also appear to increase risk [3-5].

The most common monoclonal gammopathies include MGUS, SMM, MM, Waldenström macroglobulinemia, amyloidosis light‑chain (AL), heavy‑chain disease, and other rare variants. Monoclonal gammopathy of undetermined significance is an asymptomatic premalignant condition with an approximate 1% annual risk of progression. Diagnostic criteria include serum M‑protein <3 g/dL (30 g/L), <10% clonal plasma cells in the bone marrow, absence of end‑organ damage (hypercalcemia, renal dysfunction, anemia, bone lesions (CRAB)), and exclusion of alternative causes such as amyloidosis or other hematologic malignancies [1,2]. Although typically asymptomatic, certain MGUS subtypes may predispose patients to infections, renal dysfunction, cardiovascular disease, or peripheral neuropathy due to monoclonal protein-related effects [6]. Thus, timely diagnosis and appropriate follow‑up are essential to prevent progression and irreversible complications.

Because MGUS is asymptomatic and lacks end‑organ damage, routine screening in older adults is not recommended due to the risk of false positives and unnecessary interventions [4]. Diagnostic evaluation generally includes serum creatinine, blood urea nitrogen (BUN), calcium, and total protein to monitor for progression to MM or related disorders. Serum protein electrophoresis (SPEP) and urine protein electrophoresis (UPEP) remain central to identifying and quantifying monoclonal proteins, but because these tests may miss low‑level free light chains, serum free light chain assays, immunofixation, and immunophenotypic studies are often required [7]. When abnormal proteins are detected, clinicians should assess for symptoms such as fatigue, bone pain, fractures, dyspnea, or bruising. Findings such as peripheral neuropathy, edema, lymphadenopathy, organomegaly, macroglossia, or abnormal skin changes are atypical for MGUS and may indicate MM, amyloidosis, or POEMS syndrome.

In our case, the patient’s IgG‑kappa monoclonal protein and 20% to 30% plasma cell burden initially supported a diagnosis of SMM, particularly in the absence of CRAB features. This early working diagnosis delayed recognition of POEMS, which more commonly, but not exclusively, features lambda‑restricted clones.

Atypical presentation of POEMS and diagnostic challenges

The POEMS syndrome is a paraneoplastic disorder driven by an underlying monoclonal plasma cell proliferation, most often lambda‑restricted, in which clinical manifestations arise primarily from cytokine and growth factor excess rather than tumor burden [8,9]. Vascular endothelial growth factor is the most strongly implicated mediator and is frequently elevated, with levels that often correlate with disease activity [7]. The VEGF‑mediated endothelial dysfunction and increased vascular permeability explain many hallmark features, including papilledema, vascular skin lesions, and extravascular volume overload (edema, pleural effusions, and ascites). Inflammatory cytokines likely contribute to additional multisystem involvement [7,8]. Neuropathy typically presents as a symmetric demyelinating polyradiculoneuropathy, potentially reflecting microvascular leak and inflammatory injury within peripheral nerves [7,8].

Diagnosis requires both mandatory criteria, namely polyneuropathy and a monoclonal plasma cell disorder, plus at least one major criterion (Castleman disease, sclerotic bone lesions, or elevated VEGF) and one minor criterion (organomegaly, endocrinopathy, skin changes, extravascular volume overload, papilledema, thrombocytosis, or polycythemia) [7,9]. Atypical presentations often represent incomplete or evolving phenotypes or overlap with conditions such as chronic inflammatory demyelinating polyradiculoneuropathy (CIDP), MGUS, or Castleman disease. The monoclonal protein may be low‑level and initially missed, and one or more acronym features may be absent early [7,9]. When suspicion persists despite incomplete findings, evaluation for elevated VEGF, sclerotic bone lesions, and confirmation of a plasma cell clone by biopsy becomes essential to avoid diagnostic delay [7,9].

Treatment targets the plasma cell clone and depends on the extent of the disease. Localized disease, such as solitary or limited sclerotic plasmacytoma without diffuse marrow involvement, may respond well to definitive radiotherapy [7,10]. Disseminated disease or significant marrow involvement requires systemic therapy, and eligible patients often undergo autologous stem cell transplantation (ASCT) following induction, achieving high response rates and durable survival [7]. For patients who are not ASCT candidates or require bridging therapy, immunomodulatory regimens, most commonly lenalidomide plus dexamethasone, can improve VEGF‑mediated symptoms and neuropathy while controlling the plasma cell clone [7,10]. Supportive care remains essential, including management of endocrinopathies, volume overload, thrombosis risk, and rehabilitation, with treatment response monitored clinically and supplemented by VEGF levels when available [7,10].

In our case, several findings, including thrombocytosis, lymphadenopathy, organomegaly, edema, and markedly elevated VEGF, were more consistent with POEMS syndrome than with SMM alone. Vascular endothelial growth factor is one of the most reliable biomarkers of POEMS activity and can help differentiate it from other monoclonal gammopathies [11]. Additionally, emerging evidence suggests that neuropathy in POEMS may initially be subclinical, reinforcing the importance of electrophysiological testing when clinical suspicion persists despite a normal neurological exam. Subclinical polyneuropathy was subsequently confirmed by EMG, further supporting the diagnosis.

Distinguishing MGUS, SMM, and POEMS syndrome

To contextualize the diagnostic challenge, Table 4 summarizes key differences between MGUS, SMM, and POEMS syndrome.

**Table 4:** The following tablet shows the main differences between MGUS, SMM, and POEMS MGUS: Monoclonal gammopathy of undetermined significance; SMM: Smoldering multiple myeloma; POEMS: Polyneuropathy, organomegaly, endocrinopathy, monoclonal protein, and skin changes; BMPC: Bone marrow plasma cells; CRAB: Hypercalcemia, renal dysfunction, anemia, bone lesions; VEGF: Vascular endothelial growth factor

Feature	MGUS	SMM	POEMS syndrome
Category	Premalignant plasma cell disorder	Premalignant higher-burden plasma cell disorder	Multisystem paraneoplastic syndrome due to plasma cell dyscrasia
Bone marrow plasma cells (BMPC)	<10%	10% to 60%	Usually <10% to 15%, lambda-restricted
Monoclonal protein	<3 g/dL	≥3 g/dL and/or BMPC ≥10%	Low-level monoclonal protein, typically IgA/IgG lambda
CRAB features	Absent	Absent	Not required; organ dysfunction from cytokine excess
Key diagnostic criteria	Low monoclonal protein, <10% BMPC, no end-organ damage	Monoclonal protein ≥3 g/dL and/or BMPC 10% to 60%, no CRAB/SLiM	Requires polyneuropathy + monoclonal plasma cell disorder + major/minor criteria
Major criteria (POEMS)	—	—	Sclerotic bone lesions; Castleman disease; elevated VEGF
Minor criteria (POEMS)	—	—	Organomegaly; volume overload; endocrinopathy; skin changes; papilledema; thrombocytosis/polycythemia
Clinical presentation	Asymptomatic	Asymptomatic	Progressive neuropathy, edema/ascites, endocrinopathies, skin changes
Risk of progression	~1% per year	~10% per year	Active multisystem disease, not precursor
Pathophysiology	Small stable clone	Larger active clone	VEGF-driven capillary leak + cytokine excess
Management	Observation	Close monitoring; consider trials	Treat plasma cell disorder (radiation/systemic therapy)
Prognosis	Excellent	Variable	Good with treatment; progressive if untreated

Previously reported cases

Du et al. [12] reported two cases that did not meet the criteria for typical POEMS syndrome. Both patients exhibited clinical features consistent with POEMS but lacked monoclonal protein expression, leading the treating teams to classify them as special POEMS variants. Notably, one patient had no polyneuropathy, while the other demonstrated significant polyneuropathy despite the absence of monoclonal protein. In contrast, our patient did have detectable monoclonal protein without polyneuropathy clinically; however, the EMG study showed a lack of sensory response in the right sural and superficial peroneal nerves. Both cases described by Du et al. responded favorably to standard POEMS treatment.

Tan et al. [13] reported a case that fulfilled most of the major diagnostic criteria for POEMS syndrome except for polyneuropathy. The EMG evaluation was inconclusive due to significant peripheral edema. The patient exhibited monoclonal protein and was classified as having atypical POEMS. Treatment included lenalidomide and dexamethasone followed by ASCT. These case reports highlight the need for further investigation and potentially revising the current diagnostic criteria for POEMS syndrome.

Significance of this case report

This case underscores several key diagnostic principles. First, VEGF elevation should prompt the consideration of POEMS even when the monoclonal profile is atypical. Second, subclinical neuropathy may be the earliest neurological manifestation, making EMG essential when suspicion remains high. Finally, although kappa‑restricted POEMS is uncommon, it is increasingly recognized and should not preclude diagnosis when other features strongly support the syndrome. Early identification of atypical POEMS is critical, as timely treatment, whether radiotherapy for localized disease or systemic therapy for disseminated involvement, can lead to significant clinical improvement and long‑term survival.

## Conclusions

The POEMS syndrome presents a challenging criteria categorization that needs to be met for diagnosis. Polyneuropathy, supported by EMG results, remains one of the mandatory criteria in the current nationally accepted diagnostic criteria. However, the absence of polyneuropathy clinically does not immediately eliminate the diagnosis. It is important to always consider alternative diagnoses, since a diagnosis of atypical POEMS is extremely rare. In addition, POEMS typically is lambda-restricted, but our case highlights a patient with a kappa-restricted variant, compounding this atypical presentation. This case highlights the importance of a broad differential diagnosis and early detection of atypical POEMS to ensure proper management for better outcomes.
